# *Plasmodium falciparum* Knockout for the GPCR-Like PfSR25 Receptor Displays Greater Susceptibility to 1,2,3-Triazole Compounds That Block Malaria Parasite Development

**DOI:** 10.3390/biom10081197

**Published:** 2020-08-18

**Authors:** Benedito M. dos Santos, Daniel T. G. Gonzaga, Fernando C. da Silva, Vitor F. Ferreira, Celia R. S. Garcia

**Affiliations:** 1 Department of Clinical and Toxicological Analysis, Faculty of Pharmaceutical Sciences, University of São Paulo, São Paulo-SP 05508-000, Brazil; Benedito.matheus@usp.br; 2 Fundação Centro Universitário Estadual da Zona Oeste, Unidade de Farmácia, Rio de Janeiro-RJ 23070-200, Brazil; danieltadeugonzaga@yahoo.com.br; 3 Department of Organic Chemistry, Institute of Chemistry, Universidade Federal Fluminense, Niterói-RJ 24220-900, Brazil; fcsilva@id.uff.br; 4 Department of Pharmaceutical Technology, Pharmacy School, Universidade Federal Fluminense, Niterói-RJ 24220-900, Brazil; vitorferreira@id.uff.br

**Keywords:** antimalarial, G-protein coupled receptor, heterocycles, PfSR25, *Plasmodium falciparum*, screening

## Abstract

The search for new compounds with antimalarial activity is urgent, as resistance to ones in the classical drug, has already been described in more than one continent. Compounds derived from 1,2,3-triazoles are effective against parasites and bacteria. Here, we evaluated the potential antimalarial activity against the human malaria parasite *Plasmodium falciparum* in a culture of fifty-four triazole compounds derived from 1*H*-and 2*H*-1,2,3-triazole. We identified thirty-one compounds with potential antimalarial activity at concentrations in the micromolar order (µM) and IC_50_ values ranging from 2.80 µM (**9**) to 29.27 µM (**21**). Then, we selected some of these compounds to perform the same tests on the PfSR25- strain (knockout for *P. falciparum* G-protein coupled receptor-like, SR25). Our experiences with the PfSR25- strain showed that both compounds with higher antimalarial activity for the 3D7 strain and those with less activity resulted in lower IC_50_ values for the knockout strain. The cytotoxicity of the compounds was evaluated in human renal embryonic cells (HEK 293), using MTT assays. This demonstrated that the compounds with the highest activity (**9**, **13**, **19**, **22**, **24**, **29**), showed no toxicity at the tested concentrations.

## 1. Introduction

Present in more than 90 countries, malaria is considered the most lethal infectious disease in the world. Caused by parasites of the genus *Plasmodium*, its vectors are females of the Anopheles mosquito as a vector [1]. The most recent data on the epidemiology of the disease released in 2019 by the World Health Organization estimates that in 2018 there were about 228 million cases of malaria worldwide, with more than 405,000 deaths [2].

The spread of *Plasmodium falciparum* strains resistant to commonly used drugs results in a global health crisis [3]. As a result, conventional therapies are failing and available treatment for the disease is rapidly becoming scarce [4], with classic chloroquine no longer being useful [5,6,7,8,9]. In addition, the emergence of resistance to artemisinin is increasingly common [10,11,12].

In an attempt to solve the problem generated by resistance, some approaches were used to discover new antimalarials. This includes the screening of synthetic and natural products with potential antimalarial activity [13,14]. Among them, we can mention the encapsulated synthetic protoporphyrins [15,16], hydroxynaphthoquinones [17] and indole compounds [18,19].

**Figure 1:**
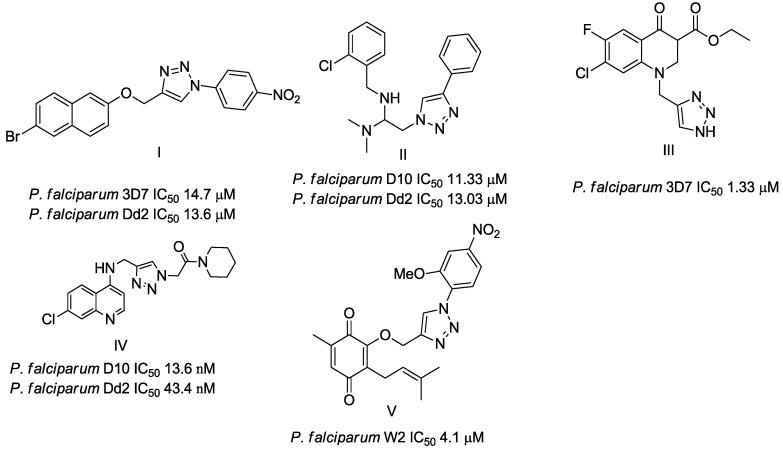
Triazole compounds with a promising antimalarial activity.

Triazole derivatives have varied pharmacological activities due to their ability to perform non-covalent interactions; they can improve solubility and the ability to bind to molecular targets of other compounds. Furthermore, the compounds 1,2,3-triazole act as a bioisosteres of different functional groups, including amide, esters, carboxylic acids and others [20,21,22,23]. Having a versatility of syntheses and applications, several derivatives of 1*H*-1,2,3-triazole entered the pharmaceutical market, among them the β-lactam-1,2,3-triazole hybrids used as antibiotics, tazobactam and cefatrizina [24]. Some 1,2,3-triazoles both as the main nucleus (I and II) and in hybrid compounds as triazole-quinolone (III), triazole-quinoline (IV), triazole-quinone (V) among others, showed promising antimalarial activity (Figure 1) [21,25,26,27,28].

Thus, the study of compounds containing the heterocycles 1*H* and 2*H*-1,2,3-triazole may lead to the discovery of promising new solutions. In the present work, we used a series of synthetic compounds derived from triazole to investigate their activity as antimalarials. In this context, our research seeks to find new drug candidates with potential antimalarial activity, our data showing that compounds **9**, **13**, **16**, **19**, **22**, **23**, **24**, **25**, **26**, and **29** showed IC_50_ values below 10 µM. The compounds with the lowest activity, **8** and **21**, showed IC_50_ values above 25 µM.

In addition, we also evaluated the activity of these compounds in the knockout strain for one of the *P. falciparum* GPCR-like PfSR25, capable of detecting changes in the K^+^ concentration coupled to Ca^2+^ signaling. More importantly, the knockout of *P. falciparum* PfSR25 similar to GPCR makes the parasite more susceptible to the antimalarial drug piperaquine [29]. PfSR25 KO is also more susceptible to nutrient hunger and ROS [29].

Here, we compare the activity of the 1*H*- and 2*H*-1,2,3-triazole heterocycles for their ability to block the 3D7 wild parasite cycle when compared to the PfSR25 knockout of *P. falciparum*. We found that both compounds with a higher antimalarial activity (**9**, **13**, **19**, **22** and **24**) for the 3D7 strain, as well as those with less activity (**21**), resulted in lower IC_50_ values for the knockout strain.

## 2. Materials and Methods 

### 2.1. Chemistry of Triazoles

The reagents used are commercial and can be purchased from several chemical companies. Specifically, common solvents, deuterated solvents and some reagents were purchased from Sigma Aldrich Brazil. The solvents were dried and distilled, and the reagents were used without purification. The silicagel column chromatography technique (Merck 70-230) was used to separate most triazoles. The development of the reactions was followed by thin-layer chromatography (TLC) on silica gel plates on 60 mesh aluminum sheets with a developer at 254 nm. The yields of triazoles refer to isolated products, purified and corroded by spectroscopic and spectrometric methods. The Thermo scientific 9100 devices were used to obtain the fusion cells. The Perkin Elmer spectrophotometer model 1420 FT-IRs was used to obtain the absorbances in the infrared region of the triazoles. It was calibrated with polystyrene at 1601.8 cm^−1^. ^1^H and ^13^C Nuclear Magnetic Resonances (NMRs) were recorded at room temperature using a Varian VXR Unity 300 or 500 MHz, in the DMSO-d_6_. Chemical shifts (δ) were given in ppm and coupling constants (J) in Hertz. The chemical shift data were reported in units of δ (ppm) downfield from solvent. The deuterated solvents were used as an internal standard. The coupling constants (J) were reported in hertz and referred to apparent peak multiplicities. High-resolution mass spectra (HRMS) were recorded on a MICROMASS Q-TOF mass spectrometer (Waters).

The methods used to prepare the 1-aryl-1*H*-1,2,3-triazoles and 2-aryl-2*H*-1,2,3-triazoles (54 examples) involved classical chemical transformations, and all evaluated compounds were from the group’s molecular bank and were present in other published works. These compounds had their infrared spectra and nuclear magnetic resonance spectra of hydrogen and carbon presented in previous works except for the compounds **20**, **21**, **22**, **25**, **26**, **29**, **52** and **54** that are now presented in the supplementary (Table S1). In our experimental conditions the compounds **52**, **53** and **54** weren’t able to be solubilized. This information can be important for future studies [30,31,32,33].

(*E*)-1-(4-chlorophenyl)-4-((2-(2,5-dichlorophenyl)hydrazono)methyl)-1*H*-1,2,3-triazole (**20**). Brown solid, 90% yield; m.p. 181–182 °C; IR (KBr, cm^−1^): ν 3215, 3142, 3077, 1594, 1534, 1488, 1455, 1423, 1357, 1269, 1202, 1134, 1079, 1036, 1012, 986, 865, 825, 785, 748, 726, 650; ^1^H MNR (500.00 MHz, DMSO-d_6_) δ: 7.03 (1H, dd, *J* 2.5 and 8.5 Hz), 7.56 (1H, d, *J* 8.5 Hz), 7.65 (1H, s), 7.79–7.85 (2H, m), 8.11–8.17 (2H, m), 9.30 (1H, s), 12.04 (1H, s); ^13^C NMR (APT, 125.0 MHz, DMSO-d_6_) δ: 112.7, 119.9, 122.4, 123.8, 125.0, 130.0, 130.7, 133.0, 133.9; 134.9, 141.9, 142.8. Anal. Calcd for C_15_H_10_Cl_3_N_5_: C, 49.14; H, 2.75; N, 19.10. Found: C, 48.74; H, 2.65; N, 19.30.

(*E*)-4-((2-(4-bromophenyl)hydrazono)methyl)-1-(3,5-dichlorophenyl)-1*H*-1,2,3-triazole (**21**). Yellow solid, 63% yield; m.p. 189–190 °C; IR (KBr, cm^−1^): ν 3275, 1581, 1526, 1485, 1438, 1399, 1288, 1262, 1172, 1116, 1068, 1045, 856, 808, 695, 665; ^1^H NMR (500.00 MHz, DMSO-d_6_) δ: 7.29–7.36 (2H, m), 7.49 (1H, s), 7.53–7.56 (2H, m), 7.95 (1H, t, *J* 2,0 Hz), 8.25 (2H, d, *J* 2.0 Hz), 9.41 (1H, s), 11.89 (1H, s); ^13^C NMR (APT, 125.0 MHz, DMSO-d_6_) δ: 111.2, 114.8, 119.3, 122.6, 123.4, 128.6, 131.8, 135.3, 137.7, 142.6, 144.0. Anal. Calcd for C_15_H_10_BrCl_2_N_5_: C, 43.83; H, 2.45; N, 17.04. Found: C, 43.43; H, 2.35; N, 17.44.

(*E*)-1-(3,5-dichlorophenyl)-4-((2-(2,5-dimethylphenyl)hydrazono)methyl)-1*H*-1,2,3-triazole (**22**). Yellow solid, 86% yield, m.p. 179–180 °C; IR (cm^−1^, filme): ν 3274, 2969, 2920, 1583, 1546, 1487, 1439, 1392, 1344, 1274, 1141, 1079, 1040, 1002, 950, 835, 793, 705, 661; ^1^H NMR (500.00 MHz, DMSO-d_6_) δ: 2.37 (3H, s), 2.39 (3H, s), 6.72 (1H, d, *J* 7.7 Hz), 7.13 (1H, d, *J* 7.7 Hz), 7.42 (1H, s), 7.44 (1H, s), 7.93 (1H, t, *J* 1.6 Hz), 8.26 (2H, d, *J* 1.6 Hz), 9.35 (1H, s), 11.47 (1H, s); ^13^C NMR de (APT, 125.0 MHz, DMSO-d_6_) δ: 16.5, 21.1, 111.8, 117.3, 119.2, 120.3, 121.0 122.8, 128.5, 130.1, 135.2, 135.9, 137.7, 142.1, 143.5. Anal. Calcd for C_17_H_15_Cl_2_N_5_: C, 56.68; H, 4.20; N, 19.44. Found: C, 56.97; H, 4.00; N, 19.80.

(*E*)-4-((2-(4-chlorophenyl)hydrazono)methyl)-1-(4-methoxyphenyl)-1*H*-1,2,3-triazole (**25**). Yellow solid, >98% yield; m.p. 223–224 °C; IR (KBr, cm^−1^): ν 3269, 3136, 2975, 2902, 2360, 1598, 1521, 1494, 1370, 1260, 1168, 1076, 1046, 905, 866, 829, 686; ^1^H NMR (500.00 MHz, DMSO-d_6_) δ: 3.97 (3H, s), 7.30 (2H, d, *J* 8.8 Hz), 7.36 (2H, d, *J* 8.8 Hz), 7.42 (2H, d, *J* 8.8 Hz), 7.48 (1H, s), 8.00 (2H, d, *J* 8.8 Hz), 9.30 (1H, s), 11.37 (1H, s); ^13^C NMR (APT, 125.0 MHz, DMSO-d_6_) δ: 55.5, 114.4, 114.9, 121.6, 122.2, 122.8, 123.3, 128.8, 129.4, 142.5, 143.6, 159.7. Anal. Calcd for C_15_H_10_Cl_2_N_6_O: C, 58.63; H, 4.31; N, 21.37. Found: C, 59.02; H, 4.27; N, 21.00.

(*E*)-1-(2,5-dichlorophenyl)-4-((2-(2,5-dichlorophenyl)hydrazono)methyl)-1*H*-1,2,3-triazole (**26**). Yellow solid, 92% yield; m.p. 200–201 °C; IR (KBr, cm^−1^): ν 3269, 3120, 2974, 2917, 1583, 1542, 1490, 1374, 1344, 1274, 1138, 1064, 1039, 1007, 950, 878, 842, 806, 711, 670, 640; ^1^H NMR (500.00 MHz, DMSO-d_6_) δ: 7.05 (1H, dd, *J* 2.4 and 8.7 Hz), 7.58 (1H, d, *J* 8.7 Hz), 7.69 (1H, d, *J* 2.4 Hz), 7.72 (1H, s), 7.91 (1H, dd, *J* 2.7 and 9.0 Hz), 7.98 (1H, d, *J* 8.4 Hz), 8.16 (1H, d, *J* 2.7 Hz), 9.14 (1H, s), 12.05 (1H, s); ^13^C NMR (APT, 125.0 MHz, DMSO-d_6_) δ: 112.7, 115.2, 119.9, 125.0, 127.6, 127.7, 128.3, 130.6, 131.9, 132.5, 132.9, 134.8, 141.9, 144.0. Anal. Calcd for C_15_H_9_Cl_4_N_5_: C, 44.92; H, 2.26; N, 17.46. Found: C, 45.12; H, 2.46; N, 17.16.

(*E*)-*N*′-((1-(3,5-dichlorophenyl)-1*H*-1,2,3-triazol-4-yl)methylene)isonicotinohydrazide (**29**) White solid, 62% yield; m.p. 194–195 °C; IR (KBr, cm^−1^): ν 2986, 1677, 1625, 1580, 1474, 1436, 1376, 1291, 1253, 1147, 1041, 985, 950, 907, 851, 808, 747, 666; ^1^H NMR (500.00 MHz, DMSO-d_6_) δ: 7.90 (1H, t, *J* 1.6 Hz), 8.10 (2H, d, *J* 5.0 Hz), 8.31 (2H, d, *J* 1.6 Hz), 8.77 (1H, s), 9.01 (2H, d, *J* 5.0 Hz), 9.60 (1H, s), 12.37 (1H, s); ^13^C NMR (APT, 125.0 MHz, DMSO-d_6_) δ: 112.2, 119.2, 119.7, 128.6, 135.6, 138.1, 141.2, 143.0, 144.4, 148.3, 161.3. Anal. Calcd for C_15_H_10_Cl_2_N_6_O: C, 49.88; H, 2.79; N, 23.27. Found: C, 50.97; H, 2.87; N, 23.00.

(*E*)-4-((2-(4-bromophenyl)hydrazono)methyl)-1-(4-methoxyphenyl)-1*H*-1,2,3-triazole (**52**). Yellow solid, >98% yield; m.p. 230–231 °C; IR (KBr, cm^−1^): ν 3268, 3136, 1591, 1518, 1489, 1369, 1301, 1257, 1174, 1110, 1066, 1030, 987, 904, 866, 829, 702, 678, 621; ^1^H NMR (500.00 MHz, DMSO-d_6_) δ: 3.97 (3H, s), 7.29-7.31 (4H, m), 7.48 (1H, s), 7.54 (2H, d, *J* 8.8 Hz), 7.99 (2H, d, *J* 8.8 Hz), 9.19 (1H, s), 11.40 (1H, s); ^13^C NMR (APT, 125.0 MHz, DMSO-d_6_) δ: 55.7, 111.0, 114.8, 115.1, 121.8, 122.4, 123.1, 129.5, 131.8, 142.6, 144.1, 145.3. Anal. Calcd for C_16_H_14_BrN_5_O: C, 58.63; H, 4.31; N, 21.37. Found: C, 58.90; H, 4.25; N, 21.10.

(*E*)-4-((2-(4-fluorophenyl)hydrazono)methyl)-1-(4-methoxyphenyl)-1*H*-1,2,3-triazole (**53**). Yellow solid, >98% yield; m.p. 195–197 °C; IR (KBr, cm^−1^): ν 3266, 3137, 2937, 2838, 1595, 1504, 1460, 1351, 1306, 1258, 1213, 1110, 1083, 1033, 905, 864, 829, 746; ^1^H NMR (500.00 MHz, DMSO-d_6_) δ: 3.97 (3H, s), 7.21-7.24 (2H, m), 7.24–7.31 (2H, m), 7.32–7.36 (2H, m), 7.43 (1H, s), 7.99–8.02 (2H, m), 9.25 (1H, s), 11.29 (1H, s); ^13^C NMR (APT, 125.0 MHz, DMSO-d_6_) δ: 55.6, 113.8, (d, *J* 7.6 Hz), 115.0, 115.6 (d, *J* 22.9 Hz), 121.6, 122.2, 122.6, 129.5, 141.4, 142.8, 155.6, 159.4 (d, *J* 215.0 Hz). Anal. Calcd for C_16_H_14_FN_5_O: C, 61.73; H, 4.53; N, 22.50. Found: C, 62.00; H, 4.45; N, 22.10.

(*E*)-4-((2-(2,5-dimethylphenyl)hydrazono)methyl)-1-(4-methoxyphenyl)-1*H*-1,2,3-triazole (**54**). Yellow solid, >98% yield; m.p. 128–130 °C; IR (KBr, cm^−1^): ν 3287, 3141, 1588, 1543, 1521, 1506, 1280, 1255, 1144, 1043, 1032, 832; ^1^H NMR (500.00 MHz, DMSO-d_6_) δ: 2.37 (3H, s), 2.39 (3H, s), 3.97 (3H, s), 6.71 (1H, d, *J* 7.3 Hz), 7.13 (1H, d, *J* 7.3 Hz), 7.30 (2H, d, *J* 8.8 Hz), 7.43 (1H, s), 7.46 (1H, s), 8.00 (2H, d, *J* 8.8 Hz), 9.19 (1H, s), 11.56 (1H, s); ^13^C NMR (APT, 125.0 MHz, DMSO-d_6_) δ: 16.6, 21.2, 55.7, 111.8, 115.1, 117.4, 120.3, 121.8, 122.4, 122.9, 129.5, 130.3, 136.0, 142.4, 143.4, 159.8. Anal. Calcd for C_16_H_14_FN_5_O: C, 67.27; H, 5.96; N, 21.79. Found: C, 67.00; H, 6.05; N, 22.00.

### 2.2. Biological Assays

All reagents used for the cell culture were obtained from Cultilab (SP-Brazil). All other reagents were of the highest possible degree on the market.

#### 2.2.1. Maintenance of *P. falciparum* Culture

Erythrocytes infected with *P. falciparum* 3D7 and the knockout strain for the PfSR25 receptor (PfSR25-) were kept in 175 cm^2^ culture bottles (Greiner Bio-One) containing RPMI 1640 with 0.5% NaHCO_3_, 0.04% sulfate gentamicin, 0.05% hypoxanthine and supplemented with 0.5% AlbuMAX I (Gibco). The culture bottles were maintained at 37 °C and an atmosphere with 90% N_2_, 5% O_2_ and 5% CO_2_ Parasitemia was determined from a blood smear stained with Panotic Rapid (Laborclin).

#### 2.2.2. Maintenance of HEK293 Cell Culture

Human Embryonic Kidney Cells (HEK293) were kept in 75 cm^2^ culture bottles (Greiner Bio-One) at 37 °C, 5% CO_2_ containing Dulbecco’s Minimal Essential Medium (DMEM), added with 10% fetal bovine serum, 1% Streptomycin and Penicillin and 3.7 g/L of NaHCO_3_ (Sigma). Cell passages were performed with trypsin-EDTA (Invitrocell). The number of cells was monitored by counting in a Neubauer chamber.

#### 2.2.3. In Vitro Growth Assay and Flow Cytometry Analysis

Red blood cells infected with *P. falciparum* (iRBC) asynchronously with 0.3% initial parasitemia and 1% hematocrit were incubated with different concentrations of compounds ranging from 0.0488 µM to 50 µM, for 72 h at 37 °C under mixture of gases. The control group was treated with solvent Dimethyl Sulfoxide (DMSO) (v/v); with the highest concentration of solvent bring 0.25%. Artemisinin was used as a positive control, with concentrations ranging from 0.15 nM to 160 nM.

Parasitemia was determined from the dot plots (side scatter versus fluorescence) of at least 103 cells acquired on an Accuri C6 flow cytometer (Becton Dickinson). Initial gating was carried out with unstained, uninfected erythrocytes to account for erythrocyte autofluorescence. The concentration responsible for 50% inhibition (IC_50_) was determined from the compound concentration-response curve determined with the software GraphPad Prism V5.01.

After 72 h, iRBCs were stained with nucleic acid marker SYBR Green I (1X) (Invitrogen) and mitochondria marker based on membrane potential MitoTracker Deep Red (50 nM) (Invitrogen), and were incubated for 20 min. at 37 °C. The final parasitemia was obtained by flow cytometer using a BD Accuri C6 Flow Cytometer, collecting 10^³^ cells. Dot plots using FlowJo V10.6.2 software determined Parasitemia. At least three independent experiments were performed in triplicate to calculate the IC_50_ values for each drug [34].

#### 2.2.4. Cytotoxicity Assay in HEK293 Cells

To evaluate the toxicity of the triazole compounds in mammalian cells, Human Embryonic Kidney cells (HEK293). Cells were plated in flat-bottom 96-well plates at a density of 104 cells per well and incubated for 24 h in 150 µL media at 37 °C, 5% CO_2_. Cells were then treated with different concentrations of each compound, ranging from 0.0488 µM to 50 µM in 200 µL DMEM for 72 h at 37 °C and 5% CO_2_. Cells treated with DMSO (v/v), with the highest concentration of solvent at 0.25% were used as control. After incubation, 40 µL of MTT reagent (solution 5 mg/mL in PBS) was added to each well, and cells were incubated for further 3 h at 37 °C and 5%CO_2_ [35]. The media was removed, and 100 µL of DMSO was added in each well. The plates were agitated in a shaker for 10 min to dissolve precipitates. The absorbance was read at 570 nm in a FlexStation 3 (Molecular Devices). Three experiments were performed independently in triplicates.

## 3. Results

**Figure 2:**
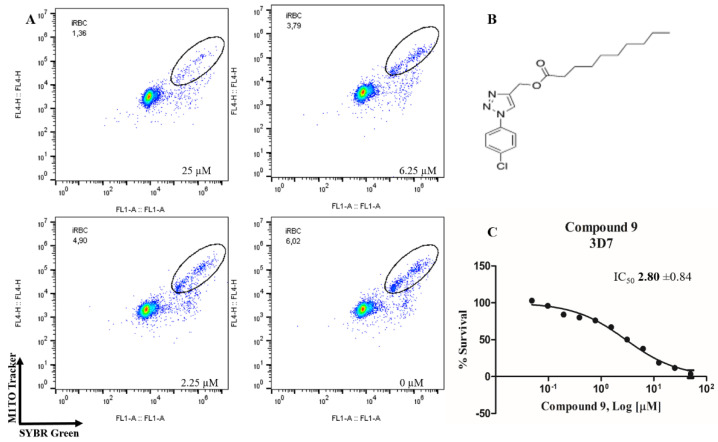
Determination of the antimalarial activity of the compound **9**. (**A**) Dot plots are shown for four different concentrations (25 µM, 6.25 µM, 2.25 µM and control) at the end of 72 h of incubation. (**B**) Chemical structure of the compound **9**. (**C**) Survival curve of the growth of *P. falciparum* in the presence of compound **9** at different concentrations (0.0488–50 µM).

In order to identify potential antimalarials present in the library of compounds derived from the 1*H*-1,2,3-triazoles and *2H*-1,2,3-triazoles, we used a culture of *P. falciparum* (3D7) to perform the screening tests of the compounds. To assess parasitemia at the end of the parasites incubation with the compounds for 72 h, we used a double-label: (1) SYBR Green I to mark DNA and (2) MitoTracker Deep Red as a mitochondria marker. The latter being dependent on the membrane potential, we ensured that only the marking and selection of viable parasites occurred. A typical analysis of parasitemia, the obtained survival and IC_50_ curves for compound 9 in 3D7 is shown in Figure 2.

Of the 54 compounds selected for screening and testing in *P. falciparum*, a total of 31 compounds showed the ability to inhibit the development of *P. falciparum* parasitemia by 50% at the tested concentrations (0.0488–50 µM). The results presented in Table 1 refer to the 31 compounds with antimalarial activity with IC_50_ values in the micromolar range. The obtaining of the IC_50_ values followed the procedure described in Figure 1, using the parasite *P. falciparum* strain 3D7. The survival and IC_50_ curves (1–31) evaluated is available in the supporting information (Figure S1).

**Figure 3:**
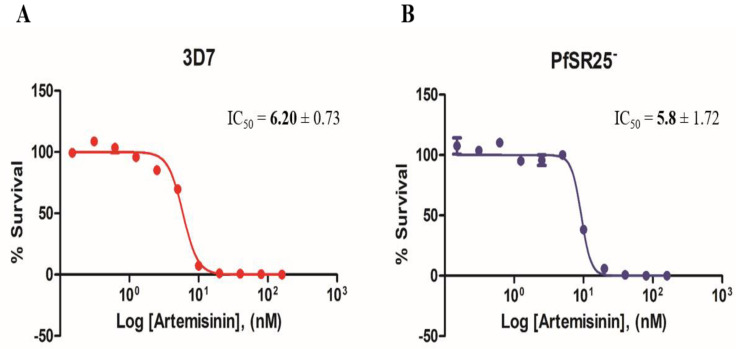
Dose-response curves for Artemisinin in *P. falciparum*. Cultures of *P. falciparum* 3D7 (**A**) and PfSR25^-^ (**B**) were incubated with artemisinin (0.15 nM to 160 nM) for 72 h. The final parasitemia was obtained using flow cytometry using double labeling with SYBR Green I and MitoTracker Deep Red.

Experiments with antimalarial artemisinin (0.15–160 nM) were performed as described for the compounds triazole as a positive control for the *P. falciparum* 3D7 strain and the PfSR25- knockout strain (Figure 3).

**Figure 4:**
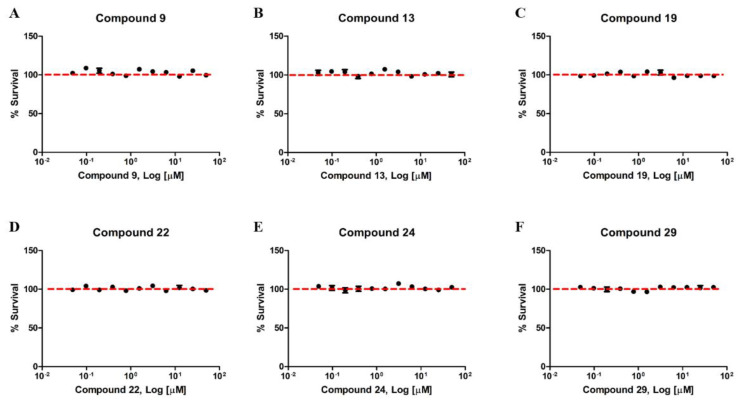
Compounds derived from the 1,2,3-triazole ring have no toxicity in HEK293 cells. Human Embryonic Kidney Cells (HEK293) were treated with different concentrations between 0.0488 µM and 50 µM of the compounds **9** (**A**), **13** (**B**), **19** (**C**), **22** (**D**), **24** (**E**) and **29** (**F**) for 72 h. The data represent the dose-response curves obtained using the GraphPad Prism software. No treatment resulted in inhibition of cell viability above 80%. Each (N = 3) independent experiment was carried out in triplicate.

The next step was to ascertain the toxicity of the compounds in HEK293 cells. For this, the compounds that showed the best antimalarial activity (9, 13, 19, 22, 24, 29) were incubated for 72 h. The results of cytotoxicity (MTT) demonstrated that none of the compounds presented toxicity in the tested concentrations (Figure 4). The observed survival values were above 80%, similar to the control group incubated with 0.25% of the DMSO.

**Figure 5:**
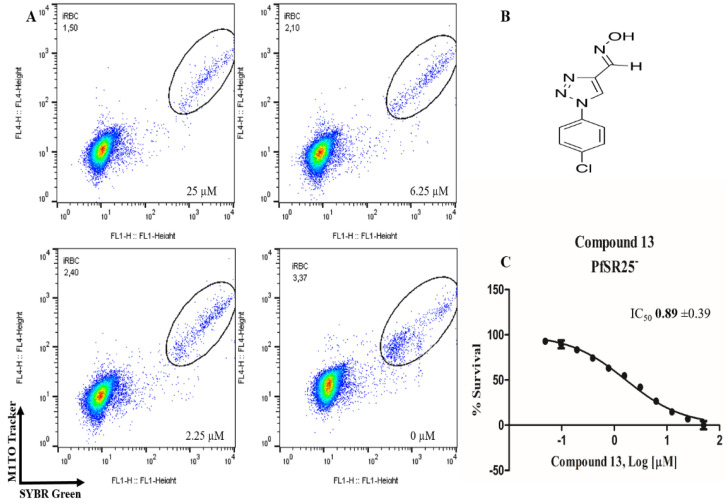
Determination of the antimalarial activity of the compound 13. (**A**) Dot plots is shown for four different concentrations (25, 6.25, 2.25 µM and control) at the end of 72 h of incubation. (**B**) Chemical structure of the compound **13**. (**C**) Survival curve of the growth of *P. falciparum* PfSR25- in the presence of the compound **13** at different concentrations (0.0488–50 µM).

We also assessed the role of PfSR25 in terms of its susceptibility to compounds with a better antimalarial activity (**9**, **13**, **19**, **21**, **22**, **24** and **29**) and compounds with less activity (**21**) based on the results obtained with the 3D7 screening. Thus, the tests were carried out like that described above and are represented in Figure 5. However, we used the knockout strain for the receptor-like PfSR25- for the tests.

The results expressed in Table 2 refer to experiments carried out with eight compounds with antimalarial activity with IC_50_ values in the micromolar range.

## 4. Discussion

*Plasmodium*, an etiological agent of malaria, is responsible for millions of death and affects the socio-economic demography in endemic areas. The parasite maturation process during the intra-erythrocytic cycle modifies the biochemistry and structural aspects of the host cells [36] and involves three distinct phases called rings, trophozoites, and schizonts [37]. One important aspect of the control of the parasite cycle is the role of melatonin and indol-derivatives. The molecules are capable of modulating the progression of the intra-erythrocytic cycle in vitro and increasing the population of schizonts [38,39].

The interruption of the parasite cell cycle progression has been the focus of the research of several labs. Regarding this, decoding the molecular aspects of parasite biology helps to tackle the drug-resistance issue drug resistance is associated with several factors [40], and in vitro resistance to piperaquine shows changes in plasmepsin 2–3 genes, which encode proteases active in the degradation of hemoglobin [38,39]. Resistance to other antimalarials such as chloroquine is also associated with genetic mutations, in this case with mutations in its transporter (PfCRT) and multiresistant protein (PfMDR1) [7,41,42,43,44].

In the present contribution, we found that most of our synthetic triazoles, when tested in vitro, were able to kill the human malaria parasite *P. falciparum* in culture assays. Cytotoxicity assays for the compounds against HEK293T cells were very low. Most active triazoles in this assay were **9**, **13**, **19**, **22**, **24** and **29** compounds. The structure of these compounds shows the presence of the groups nonyl, 4-fluorophenyl, 2,5-dichlorophenyl, phenyl and isocotinyl, which may be associated with a higher efficiency of groups as antimalarials; this requires further investigation. It is important to note that, with the exception of ester 9, all active compounds showed the C=N bond, which shows that this must have some relationship with antimalarial activity.

Molecules that presented groups linked in the N-1 position were more active when compared with groups linked in the N-2 position. This geometry probably favors a greater interaction with the receptor GPCR-like PfSR25. The most active compounds had the chlorine atom and methoxy group in position C-4 (compounds **9**, **13**, **19**, and **24**) thus, an electronegative group in that position is important for antimalarial activity. The comparison between the compounds **24** and **29** reveals that the chlorine atom at the C-3 and C-5 positions decreases antimalarial activity tenfold. The group in position C-4 of the triazole ring also seems important when comparing compounds **13** and **19**.

Based on previous docking studies on different target proteins such as protein kinase inhibitors by Limonoids from *Azadirachta indica* [45], inhibition of dihydroorotate dehydrogenase (PfDHODH) by indolyl-3-ethanone-α-thioethers derivatives [46], inhibition of *Plasmodium falciparum* methionine aminopeptidase 1 by pyrimidine derivatives [47] and others, we intend to carry out docking studies to prove our hypotheses. These studies will be guided towards a defined target: the GPCR-like PfSR25 receptor. In addition, the objective is to propose new molecules that can be more active against Plasmodium falciparum.

We reported that *Plasmodium* displays four G protein-coupled receptor-like (GPCR-like) proteins: PfSR1, PfSR10, PfSR12 and PfSR25 [48]. These candidates were identified by using bioinformatics as potential serpentine receptors due to a typical protein signature that contains seven transmembrane domains.

The serpentine-like receptor PfSR25 is a parasite sensor of a shift in the potassium concentration. Of note, *P. falciparum* faces a distinct environment due to its life cycle, including ionic changes between the bloodstream and host cell interior. The biological process that encompasses these changes includes parasite invasion and egress from host cells. The molecular mechanism for PfSR25 action identified that changes in [K+] were linked to the parasite cytosolic changes in Ca^2+^. The study identified the PfSR25 knockout strain as being more susceptible to stress and antimalarial action when compared to the wild type strain [29].

We have therefore have used the PfSR25 knockout parasite strain in culture to investigate the most active 1,2,3-triazole compounds, based on the results of the screening in the 3D7 *P. falciparum* assays. Our results demonstrate the susceptibility of the knockout to this class of promising antimalarials derived from triazole. Based on our findings and previous studies published by our group using the knockout strain for the SR25 receptor [29], we believe that the SR25 receptor should be related to drug resistance. Thus, the absence of the receptor increases the susceptibility of the PfSR25- strain to some classes of antimalarials.

## 5. Conclusions

The results demonstrated here allow us to conclude that comparative screening studies using *P. falciparum* WT and knockout for PfSR25 are essential for dissecting the mechanism of action and potential participation of this receptor in the resistance to antimalarials in *P. falciparum*. These studies open new frontiers as they allow us to explore aspects of the parasite’s biology by dissecting the GPCR-like receptors’ role, the K^+^ sensor (PfSR25) and the mechanism of action of drugs in *P. falciparum*. In addition to this, the studies may represent the identification of 1*H*-1,2,3-triazoles and 2*H*-1,2,3-triazoles as potential antimalarials.

## Figures and Tables

**Table 1:**
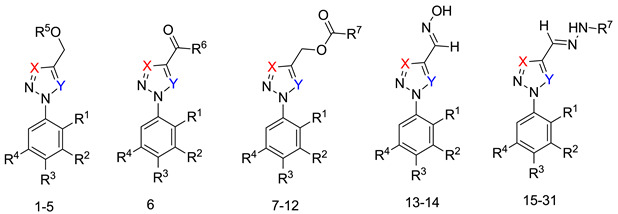
Results obtained with the screening of the 1*H*-1,2,3-triazoles library in *P. falciparum* (3D7); molecular structure of each compound; IC_50_ values with the standard error of the mean.

Compound	R^1^	R^2^	R^3^	R^4^	R^5^	R^6^	R^7^	R^8^	X	Y	IC_50_ (μM)
**1**	H	H	Cl	H	Et	-	-	-	N	CH	11.65 ± 0.74
**2**	H	Cl	H	Cl	Et	-	-	-	N	CH	15.41 ± 2.26
**3**	H	Cl	H	Cl	Pr	-	-	-	N	CH	14.52 ± 1.71
**4**	Cl	H	H	Cl	Pr	-	-	-	N	CH	20.45 ± 0.73
**5**	H	Cl	H	Cl	Bu	-	-	-	N	CH	23.94 ± 2.01
**6**	H	H	H	H	-	OEt	-	-	N	CH	12.84 ± 3.02
**7**	H	H	H	H	-	-	Ph	-	N	CH	22.93 ± 0.34
**8**	H	H	H	H	-	-	Ph	-	CH	N	27.49 ± 1.26
**9**	H	H	Cl	H	-	-	nonyl	-	N	CH	2.80 ± 0.84
**10**	H	Cl	H	Cl	-	-	nonyl	-	N	CH	14.64 ± 0.29
**11**	H	H	OMe	H	-	-	nonyl	-	N	CH	23.46 ± 2.77
**12**	H	H	OMe	H	-	-	Ph	-	N	CH	13.53 ± 1.12
**13**	H	H	Cl	H	-	-	-	-	N	CH	6.67 ± 1.86
**14**	Cl	H	H	Cl	-	-	-	-	N	CH	21.52 ± 0.84
**15**	H	H	H	H	-	-	-	Ph	CH	N	18.05 ± 1.92
**16**	H	H	H	H	-	-	-	4-Cl-Ph	CH	N	9.29 ± 1.01
**17**	H	H	H	H	-	-	-	4-F-Ph	CH	N	14.68 ± 1.28
**18**	H	H	Cl	H	-	-	-	4-Cl-Ph	N	CH	17.61 ± 1.07
**19**	H	H	Cl	H	-	-	-	4-F-Ph	N	CH	8.45 ± 2.25
**20**	H	H	Cl	H	-	-	-	2.5-diCl-Ph	N	CH	13.58 ± 1.20
**21**	H	Cl	H	Cl	-	-	-	4-Br-Ph	N	CH	29.27 ± 0.34
**22**	H	Cl	H	Cl	-	-	-	2,5-diCl-Ph	N	CH	5.56 ± 0.47
**23**	H	Cl	H	Cl	-	-	-	2,5-diMe-Ph	N	CH	9.23 ± 1.68
**24**	H	H	OMe	H	-	-	-	Ph	N	CH	4.44 ± 1.43
**25**	H	H	OMe	H	-	-	-	4-Cl-Ph	N	CH	9.00 ± 3.27
**26**	Cl	H	H	Cl	-	-	-	2,5-diCl-Ph	N	CH	9.40 ± 2.87
**27**	H	H	H	H	-	-	-	-CO-4-pyridil	N	CH	23.12 ± 2.58
**28**	H	H	Cl	H	-	-	-	-CO-4-pyridil	N	CH	14.52 ± 1.13
**29**	H	Cl	H	Cl	-	-	-	-CO-4-pyridil	N	CH	6.96 ± 1.44
**30**	H	H	OMe	H	-	-	-	-CO-4-pyridil	N	CH	23.20 ± 1.94
**31**	H	H	H	H	-	-	-	-CO-4-pyridil	CH	N	20.16 ± 0.79

**Table 2:** Results obtained with the screening of compounds in the knockout *P. falciparum* for the PfSR25 (PfSR25-) receptor; molecular structure of each compound; IC_50_ values with the standard error of the mean.

Compound	Molecular Structure	IC_50_ µM
**9**	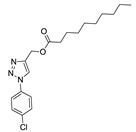	**1.55** ± 0.55
**13**	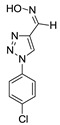	**0.89** ±0.39
**19**	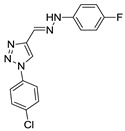	**2.24** ± 0.77
**21**	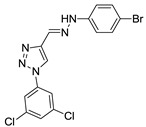	**12.42** ± 2.51
**22**	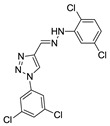	**4.41** ± 0.60
**24**	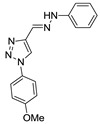	**0.99** ± 0.25
**29**	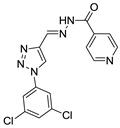	**9.91** ± 2.94

## Supplementary Materials

The following are available online at https://www.mdpi.com/2218-273X/10/8/1197/s1, Figure S1: Growth curve of *P. falciparum* in the presence of compounds (**1**–**31**) in different concentrations (0.0488–50 μM). Table S1: Compounds (**1**–**54**) that have demonstrated antimalarial activity **1**–**31** and compounds without the ability to inhibit the development of *P. falciparum* parasitemia by 50% at the tested concentrations (0.0488–50 μM) **32**–**54**.
