# Supplementary material for: Plasmodium falciparum Knockout for the GPCR-Like PfSR25 Receptor Displays Greater Susceptibility to 1,2,3-Triazole Compounds That Block Malaria Parasite Development
Source: Biomolecules. 2020 Aug 18;10(8):1197. doi: 10.3390/biom10081197 (PMC7465636; doi:10.3390/biom10081197)
Supplement: Supplementary file 1 [file biomolecules-10-01197-s001.pdf]

Supplementary Table (S1)

1-31 Compounds that have demonstrated antimalarial activity.

32-54 Compounds without the ability to inhibit the development of *P. falciparum* parasitemia by 50% at the tested concentrations (0.0488 - 50 µM).

Compound	R^1	R^2	R^3	R^4	R^5	R^6	R^7	R^8	X	Y	IC_{50} (µM)
**1**	H	H	Cl	H	Et	-	-	-	N	CH	11.65 ± 0.74
**2**	H	Cl	H	Cl	Et	-	-	-	N	CH	15.41 ± 2.26
**3**	H	Cl	H	Cl	Pr	-	-	-	N	CH	14.52 ± 1.71
**4**	Cl	H	H	Cl	Pr	-	-	-	N	CH	20.45 ± 0.73
**5**	H	Cl	H	Cl	Bu	-	-	-	N	CH	23.94 ± 2.01
**6**	H	H	H	H	-	OEt	-	-	N	CH	12.84 ± 3.02
**7**	H	H	H	H	-	-	Ph	-	N	CH	22.93 ± 0.34
**8**	H	H	H	H	-	-	Ph	-	CH	N	27.49 ± 1.26
**9**	H	H	Cl	H	-	-	nonyl	-	N	CH	2.80 ± 0.84
**10**	H	Cl	H	Cl	-	-	nonyl	-	N	CH	14.64 ± 0.29
**11**	H	H	OMe	H	-	-	nonyl	-	N	CH	23.46 ± 2.77
**12**	H	H	OMe	H	-	-	Ph	-	N	CH	13.53 ± 1.12
**13**	H	H	Cl	H	-	-	-	-	N	CH	6.67 ± 1.86
**14**	Cl	H	H	Cl	-	-	-	-	N	CH	21.52 ± 0.84
**15**	H	H	H	H	-	-	-	Ph	CH	N	18.05 ± 1.92
**16**	H	H	H	H	-	-	-	4-Cl-Ph	CH	N	9.29 ± 1.01
**17**	H	H	H	H	-	-	-	4-F-Ph	CH	N	14.68 ± 1.28
**18**	H	H	Cl	H	-	-	-	4-Cl-Ph	N	CH	17.61 ± 1.07
**19**	H	H	Cl	H	-	-	-	4-F-Ph	N	CH	8.45 ± 2.25
**20**	H	H	Cl	H	-	-	-	2.5-diCl-Ph	N	CH	13.58 ± 1.20
**21**	H	Cl	H	Cl	-	-	-	4-Br-Ph	N	CH	29.27 ± 0.34
**22**	H	Cl	H	Cl	-	-	-	2,5-diCl-Ph	N	CH	5.56 ± 0.47
**23**	H	Cl	H	Cl	-	-	-	2,5-diMe-Ph	N	CH	9.23 ± 1.68
**24**	H	H	OMe	H	-	-	-	Ph	N	CH	4.44 ± 1.43
**25**	H	H	OMe	H	-	-	-	4-Cl-Ph	N	CH	9.00 ± 3.27
**26**	Cl	H	H	Cl	-	-	-	2,5-diCl-Ph	N	CH	9.40 ± 2.87
**27**	H	H	H	H	-	-	-	-CO-4-pyridil	N	CH	23.12 ± 2.58
**28**	H	H	Cl	H	-	-	-	-CO-4-pyridil	N	CH	14.52 ± 1.13
**20**	H	Cl	H	Cl	-	-	-	-CO-4-pyridil	N	CH	6.96 ± 1.44
**30**	H	H	OMe	H	-	-	-	-CO-4-pyridil	N	CH	23.20 ± 1.94
**31**	H	H	H	H	-	-	-	-CO-4-pyridil	CH	N	20.16 ± 0.79
**32**	H	H	Cl	H	Bu	-	-	-	N	CH	ND
**33**	H	H	Cl	H	Pr	-	-	-	N	CH	ND
**34**	Cl	H	H	Cl	-	-	Me	-	N	CH	ND
**35**	H	H	Cl	H	-	-	Me	-	N	CH	ND
**36**	Cl	H	H	Cl	-	-	Ph	-	N	CH	ND
**37**	Cl	H	H	Cl	-	-	pentyl	-	N	CH	ND
**38**	H	H	Cl	H	-	-	pentyl	-	N	CH	ND
**30**	H	H	Cl	H	-	-	Ph	-	N	CH	ND
**40**	H	H	OMe	H	-	-	nonyl	-	N	CH	ND
**41**	H	H	OMe	H	-	-	pentyl	-	N	CH	ND
**42**	H	Cl	H	Cl	-	-	pentyl	-	N	CH	ND
**43**	H	Cl	H	Cl	-	-	Me	-	N	CH	ND
**44**	H	Cl	H	Cl	-	-	Ph	-	N	CH	ND

**45**	H	H	H	H	-	-	nonyl	-	N	CH	ND
**46**	H	Cl	H	Cl	-	-	-	-	N	CH	ND
**47**	H	H	H	H	-	-	-	4-F-Ph	N	CH	ND
**48**	H	H	H	H	-	-	-	4-Br-Ph	N	CH	ND
**49**	H	H	Cl	H	-	-	-	Ph	N	CH	ND
**50**	H	H	Cl	H	-	-	-	4-Br-Ph	N	CH	ND
**51**	H	Cl	H	Cl	-	-	-	4-Cl-Ph	N	CH	ND
**52**	H	H	OMe	H	-	-	-	4-Br-Ph	N	CH	ND
**53**	H	H	OMe	H	-	-	-	4-F-Ph	N	CH	ND
**54**	H	H	OMe	H	-	-	-	2,5-diMe-Ph	N	CH	ND
